# Concurrent infection of dengue virus with malaria parasites among outpatients attending healthcare facilities in Benin city, Nigeria

**DOI:** 10.1097/j.pbj.0000000000000249

**Published:** 2024-04-15

**Authors:** Joy Zitgwai Saidu, Rachel Obhade Okojie

**Affiliations:** Department of Microbiology, Faculty of Life Sciences, University of Benin, Benin City, Edo State, Nigeria

**Keywords:** dengue virus, malaria parasites, serology, molecular, DENV serotype 2

## Abstract

**Background:**

Dengue virus (DENV) and malaria parasites (MP) are among the common febrile diseases affecting the tropics and subtropics of the world. Both are mosquito-borne pathogens affecting humans and other animals.

**Methods:**

Blood samples were collected from 280 consented out-patients attending the selected hospitals and were analyzed. Malaria parasites were detected using microscopy and Malaria Ag Pf/Pan Rapid Test Device. Dengue virus was detected by serology and heminested reverse transcriptase PCR (hnRT-PCR) to target the flavivirus polymerase (NS5) gene.

**Results:**

Malaria parasites recorded a total positivity of 151 patients (53.9%) using microscopy, while DENV antibodies (DENV IgM and DENV IgG) were positive in 16 (5.7%) and 39 (13.9%) patients, respectively. There was a concurrent infection between MP/DENV IgM in 13 (4.6%) patients and MP/DENV IgG in 27 (9.6%) patients. Molecular identification revealed DENV serotype 2 in circulation.

**Conclusion:**

This study documents molecular evidence of dengue virus coexisting with malaria parasites in the study population, hence the need for efficient surveillance and control system.

## Introduction

Malaria is a major public health problem in tropical and subtropical countries and a mosquito-borne infectious disease of humans and other animals caused by protozoans of the genus *Plasmodium.*^1,2^ The World Health Organization has estimated that 216 million documented cases of malaria and around 655,000 people died from the disease in 2010, many of whom were children younger than 5 years.^3^ Dengue fever (DF) is one of the most common and important arboviral and febrile diseases in the tropical and subtropical regions of the world.^4,5^ In 2011, World Health Organization (WHO) reported that about 2.5 billion people are at risk of dengue infection globally, whereas 50 million dengue virus infection occurs annually.

Nigeria, especially southern part, is characterized by a hot and humid weather, where the rainfall is long lasting from May to November with average annual rainfall of 250 mm. *Aedes aegypti* and *A. albopictus* are domestic mosquitoes that are known to be the major vectors of dengue viruses in the tropics and subtropics.^6^ In recent decades, the incidence and geographical expansion of dengue virus have increased worldwide due to climate change, virus evolution, lack of vector surveillances or deteriorating vector control, increasing population, mobility and uncontrolled urbanization which substantially increase the density, larval development, and survival of the vector.^7–9^

People in the tropic and subtropic areas are at a risk of contracting both infections concurrently.^10,11^ In Nigeria, there has been quite a number of studis on concurrent dengue and malaria but there are very little information regarding molecular and epidemiological studies of circulating dengue virus; we therefore aimed to ascertain the prevalence and molecular characteristics of dengue virus coexisting malaria among outpatients presenting at healthcare facilities in Benin City, Nigeria.

## Methods

### Study design

The study was a population-based, descriptive cross-sectional design performed in Benin City, Edo state, southern part of Nigeria. Benin City, the capital of Edo state, with population of about 1,147,188, density of 1,200/km^2^ and lies geographically on 6°20'00"N 5°37'00"E with a land mass of 19,794 km^2^. The city has a humid climate and two climate seasons: the rainy and dry season. The rainy season is between April and October with average rainfall of 250 cm. The dry season lasts from November to March and also a cold humid and dusty Harmattan period between December and January. The average temperature ranges between 22°C in the rainy season and 28°C in the dry season.^12^

The study population was both adults and children of all age groups and both sexes who came for outpatient consultation at Central Hospital Sapele Road and St. Philomena Hospital Dawson Road health facilities. Consecutive consented participants were recruited, and a structured questionnaire was provided to each consented patient to obtain demographic and risk factors. The study was performed from July 2019 to February 2020.

### Ethical considerations

Ethical clearance was duly obtained from Research and Ethics Committee Edo state Ministry of Health with reference number HA.577/Vol.11/18/156. Informed consent was obtained from either the participants or their guardians (in the case of minors) before the recruitment. Participation was voluntary, and the cost of the tests was not being borne by the participants.

### Sample collection and processing

From the two hundred and eighty consented participants, a total of 5 mL venous blood were collected from each participant, of which 2 mL of each blood sample was decanted in a plain bottle; of these, half of the blood was used to perform the malaria Rapid Diagnostic Test (RDT) on site and prepare thick blood films for microscopy. The remaining blood was immediately centrifuged at 5000 rpm for 10 minutes to obtain serum for dengue virus screening using the DENV IgM/IgG rapid diagnostic kits. The remaining 3 mL of blood was stored in RNA shield solution for molecular characterization of dengue virus. All samples for further studies were stored at −20°C.

### Microscopy

Microscopy was performed using thick film: Two drops of blood were placed on a clean glass slide and smeared in a circular motion over an area of about 2 cm in diameter. The films were allowed to air-dry at room temperature. The thick films were covered with 3% Giemsa stain for 15–30 minutes and subsequently washed with clean water and allowed to air-dry and observed under the microscope using x100 oil immersion magnification, as described by Cheesbrough 2010.^13^

### Rapid test method

In addition, Malaria Ag Pf/Pan Rapid Test Device (Whole Blood) (LUGENE China) was used for malaria diagnosis. The Malaria Ag Pf/Pan antigen rapid test is a qualitative and rapid chromatographic immunoassay for the differential diagnosis of *Plasmodium falciparum* histidine-rich protein II (P.f HRP-II) and antialdolase antibodies common to *P falciparum*, *P. malaria*, *P. ovale*, *and P. vivax*, as described by Achonduh-Atijegbe et al.^14^

### Rapid diagnostic testing for dengue virus

Diagnosis of dengue virus was performed using the dengue virus IgG/IgM RDT (LUGENE China). It is a chromatographic immunoassay that can detect and differentiate IgG and IgM antibodies to dengue virus in human blood. Two drops of serum were added to the sample followed by two drops of buffer. The results were read in 15 minutes. The appearance of a colored band in the control (C) line and in the M or/and G lines was considered as positive for IgM or/and IgG antibodies, respectively, and the absence of a color band in both the G and M lines but present only in the C was considered negative, as described by Lee et al.^15^

### Molecular identification of dengue virus

Blood specimens (in RNA shield) that were positive to dengue virus using rapid test devices were subjected to RNA extraction. The viral RNA was extracted with Zymo Quick-RNA^TM^ viral extraction kits (CAT# R1034 & R1035) according to manufacturer's instruction. The extracted RNA was reverse transcript using One Taq® RT-PCR kit (BioLabs, New England). Hemi-nested reverse transcriptase PCR (hnRT-PCR) with the generic primers; FLAVMAMD/FLAVCFD2/FLAVFS778 targeting highly conserved motifs in the flavivirus polymerase (NS5) gene (280-bp) as described by Scaramozzino et al^16^ with little modifications. A total volume of 40 µL containing 20 µL of one Taq Hot start (1x) Master Mix 0.8µl of Forward primer FLAVMAMD (AACATGATGGGRAARAGRGARAA), 0.8 µL of reverse primer FLAVCFD2 (GTGTCCCAGCCGGCGGTGTCATCAGC), and 15.4 µL of nuclease-free H_2_O and then 3 µL of cDNA were added to each mixture. The reaction conditions were as follows: primary amplification: 94°C for 5 minutes and then 25 cycles of 94°C for 1 minute, annealing: 53°C for 1 minute, extension: 72°C for 1 minute, and final extension at 72°C for 5 minutes. The second step of the heminested PCR was performed using 0.8 µL of FLAVCFD2 primer, 0.8 µL FLAVFS778 (AARGGHAGYMCDGCHATHTGGT) primer, Taq Hot start (1x) Master Mix of 20 µL, 15.4 µL of nuclease-free H_2_O, and 1 µL of the first PCR product was added as template. The mixture was amplified as follows: primary amplification: 94°C for 5 minutes and then 30 cycles of 94°C for 1 minute, annealing: 54°C for 1 minute, extension: 72°C for 1 minute, and final extension at 72°C for 5 minutes. After that, the amplicons were identified by their molecular weight analyzed by electrophoresis. Sequences obtained were edited and aligned with ClustalW, and the phylogenetic tree was constructedusing the neighbor-joining method (MEGA 6) with bootstrap 500 replications. The evolutionary distances were computed using the Maximum Composite Likelihood method.

### Data analysis

Data collected from participants were analyzed using SPSS version 20.0 statistical software. Descriptive analysis and proportions were calculated for categorical data and to obtain prevalence. Pearson's logistic regression was used to examine the association between some factors and dengue virus infection; with calculation of odds ratios (OR) and 95% confidence intervals (CI), this was performed. Mean age was also expressed in standard error, statistical significance was determined with the chi-square test, and the *P* value was set at 0.05.

## Results

In this study, 280 participants were enrolled; the mean age of the population was 37 (±12.7) years. Among the participants, 93 (33.2%) were male and 187 (66.8%) were female. The presence of symptoms recorded were headache (45.4%), fever (42.5%), cold (26.1%), joint pains (38.6%), and rashes (2.5%). In total, 57.5% of patients reported sleeping under insecticide treated bed net (ITN), while 83.2% stored water in open and close containers inside and outside their homes. In addition, 13.6% of the participants were under antimalarial drugs before presenting to the hospital. The characteristics of the participants of the study are presented in Table 1. The odds ratio analysis revealed that participants who sleeps under an ITN are not protected from the DENV infection (OR: 1.158, 95 % CI: 0.629–2.130, *P*: 0.068) as compared with those who did not use an ITN. There was no association between dengue virus infection with water storage inside and outside houses (OR: 1.727, 95% CI: 0.692–4.309, *P*: 0.071) and blood transfusion (OR: 0.672, 95% CI: 0.569–4.909, *P*: 0.347).

**Table 1:** Characteristics and DENV IgM occurrences of the study population (N = 280).

Variables		Participants at enrollment	Positive to DENV IgM	*P* value	Odds ratio (95 % CI)
Mean age (years ±standard error)		(37 ± 12.7)			
Gender	Males	93 (33.2%)			
	Females	187 (66.8%)			
Headache	Yes	127 (45.4%)	10 (3.6)	.156	2.094 (0.740–5.929)
	No	153 (54.6%)	6 (2.1)		
Fever	Yes	119 (42.5%)	10 (3.6)	.096	2.370 (0.837–6.714)
	No	161 (57.5%)	6 (2.1)		
Cold	Yes	73 (26.1%)	6 (2.1)	.284	1.764 (0.618–5.038)
	No	207 (73.9%)	10 (3.6)		
Joint pains	Yes	108 (38.6%)	8 (2.9)	.239	1.640 (0.597–4.507)
	No	172 (61.4%)	8 (2.9)		
Rashes	Yes	7 (2.5%)	1 (0.4)	.322	2.867 (0.324–25.361)
	No	273 (97.5%)	15 (5.4)		
Use of insecticide treated net (ITN)	Yes	161 (57.5%)	12 (4.3)	.068	1.158 (0.629–2.130)
	No	119 (42.5%)			
Water storage indoors and outdoors	Yes	233 (83.2%)	14 (5.0)	.071	1.727 (0.692–4.309)
	No	47 (16.8%)			
Blood transfusion	Yes	18 (6.4%)	1 (0.4)	.347	0.672 (0.569–4.909)
	No	262 (93.6%)			
Antimalaria used	Yes	38 (13.6%)	3 (1.1)	.533	1.510 (0.410–5.567)
	No	242 (86.4%)			

95% CIs based on likelihood method.

CI, confidence interval; N, number.

The prevalence of malaria by microscopy and RDT was 53.9% and 15%, respectively, while the prevalence of DENV IgM and DENV IgG was 5.7% and 13.9%, respectively. Seropositivity and coexistence of DENV IgM and IgG was 18.9% (95% CI: 0.05–3.09). Coinfection of malaria by microscopy with DENV IgM and DENV IgG was 2.6% (95% CI: 0.89–1.04), whereas there was no coinfection of malaria by mRDT with DENV IgM and DENV IgG (Table 2).

**Table 2:** Prevalence and coexistence of dengue virus and malaria.

	N	% (95% CI)
Disease test		
MP microscopy	151	53.9 (1.40–1.52)
MP RDT	42	15 (1.85–1.89)
DENV IgM	16	5.7 (1.92–1.97)
DENV IgG	39	13.9 (1.82–1.90)
DENV IgM and DENV IgG	53	18.9 (1.76–1.86)
Coinfection with malaria		
MP microscopy and DENV IgM	13	4.6 (1.10–14.21)
MP microscopy and DENV IgG	27	9.6 (1.03–4.39)
MP microscopy and DENV IgM and IgG	1	2.6 (0.89–1.04)
MP RDT and IgM	5	1.8 (0.92–8.49)
MP RDT and IgG	6	2.1 (0.41–2.65)
MP RDT and IgM and IgG	0	0

95% CIs based on likelihood method.

CI, confidence interval; N, number; DENV, dengue virus; MP, malaria parasites; RDT, Rapid diagnostic test; IgM, immunoglobulin M; IgG, Immunoglobulin G.

Based on the results presented in Table 3, malaria microscopy was considered as the gold standard for malaria parasites identification. The RDT sensitivity was 57.9%, specificity was 99.2%, positive predictive value was 27.2%, and the negative predictive value was 99.2%. The measurement of agreement (Kappa) value was 0.248 with *P* value of 0.000.

**Table 3:** Comparison of malaria RDT and microscopy (gold standards) for the detection of malaria parasites.

	Microscopy								
		Positive (%)	Negative (%)	Sensitivity (%)	Specificity (%)	PPV (%)	NPV (%)	Kappa	*P* value
RDT	Positive	41 (14.6)	1 (0.4)	57.9	99.2	27.2	99.2	.248	0.000
	Negative	110 (39.3)	128 (45.7)						

RDT, rapid diagnostic value; %, percentage; PPV, positive predictive value; NPV, negative predictive value; Kappa, degree of agree.

**Figure 1.**
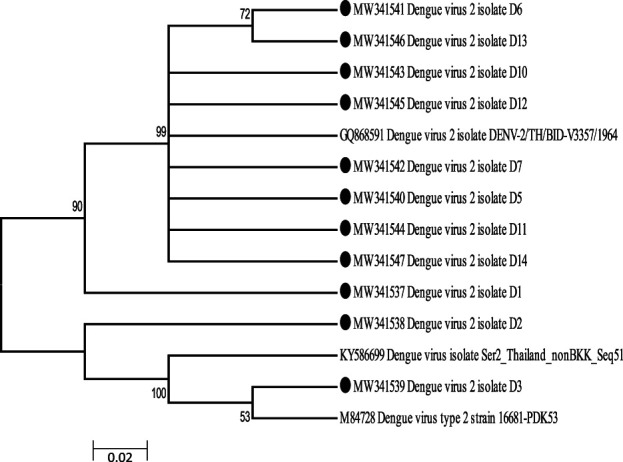
Phylogenetic analysis of dengue virus by neighbor-joining method using MEGA 6.

The gene sequences of eleven DEN-2 that were aligned with other strains were used to construct phylogenetic tree (Fig. 1). The phylogenetic tree is based on the complete E gene sequences and constructed using the neighbor-joining method (MEGA 6) with bootstrap 500 replications. Those indicated in solid circles are those isolated in this research and were aligned with other strains. The tree shows that isolate D1, D5, D7, D10, D11, D12, and D14 are closely related and formed a clade with D6 and D13. Also, isolate D3 clustered with dengue virus type 2 strain 16681 and formed a clade with dengue virus from Thailand.

## Discussion

The overall prevalence of malaria was 151 patients (53.9%) using microscopy and 42 patients (15%) using the RDT. The prevalence of 53.9% using microscopic method is higher than 43.1% reported in Rivers,^17^ 43.4% in Cameroon,^14^ and 40.5% in South Eastern Nigeria.^18^ The prevalence of 42 patients (15%) using malaria RDT is seen to be lower than 35.6% reported in Kaduna^19^ and 21.6% from children younger than 5 years in Ibadan.^20^ The comparison of malaria RDT with microscopic method in the diagnosis of malaria parasites status in this research was performed. Microscopy performed better than the rapid diagnostic test kit. Microscopy, using Giemsa method, is regarded as the most suitable and gold standard for malaria diagnosis because it is inexpensive to perform, able to differentiate malaria species, and even quantify malaria parasites.

In this study, the RDT sensitivity of 57.9% recorded is seen to be lower than 94.6% by Adebisi et al,^20^ 94.3% by Falade et al,^21^ and other values varying from 100%^22^ to 42.3%.^23^ The observed differences of such sensitivity may be due to the functionality of RDT which is unable to detect parasites at low density (<200–400 µl). Also, other factors responsible for the disparity might be storage of the kits. The manufacturer's recommended temperature of 2–30°C for storage of histidine-rich protein-based tests, these might have contributed to the different sensitivity.^24^

Dengue fever in Nigeria is likely to go untreated or misdiagnosed as malaria or referred to as fever of unknown etiology. The incorporation of dengue virus diagnosis into febrile illnesses diagnosis is important to provide appropriate treatment and management of the patients and to prevent potential dengue outbreak. Nigeria is among the tropical countries in which dengue is reported to be endemic.^25^ However, it is likely that many cases of dengue in Nigeria are often undiagnosed or misdiagnosed as malaria or other fever of unknown cause. It is said that many outbreaks have been neglected, unrecognized, and underreported due to unavailability of diagnostic tools and staff unawareness in health centers.^26^

The use of dengue virus rapid test kit is of importance; it is designed to detect both dengue virus NS1 antigen and IgM/IgG antibodies in human blood, which has made dengue virus diagnosis possible as early as Day 1 of dengue virus infection.^27^ The rapid tests for dengue are relatively inexpensive, quick, simple, and easy to perform. This point-of-care lateral flow assay for dengue has been validated for use in dengue endemic areas,^28,29^ as it fulfills the World Health Organization (WHO) Affordable, Sensitive, Specific, User-friendly, Rapid & Robust, Equipment-free, and Delivered (ASSURED) criteria for point-of-care testing.

Concurrent infection with malaria and DENV has previously been reported.^30,31^ Research has shown that coexistence of arboviruses with malaria may result in more severe clinical manifestations.^32,33^ Nigeria is one of the few African countries that limit investigation of febrile illnesses to malaria and typhoid with complete neglect to other febrile pathogens such as viral infections. Generally, viral infections suppress the natural immunity of the host, and this often allows opportunistic infections to set in.^34^ Therefore, a concurrent infection of DENV and malaria as observed in this study is a pointer to the fact that coinfections occurred.

The usage of mosquito nets has been identified as the most effective malaria control approach and other mosquito-borne pathogens.^35^ There was link between mosquito net use and DENV antibodies in this group (*P* < 0.05). The use of mosquito nets within this cohort did not reach the malaria control targets set in an endemic area, that is, it fell short of the global aim of >80% of individuals in at-risk groups using them.^36^ Long-lasting insecticide-treated nets have been demonstrated to dramatically prevent the transmission of malaria parasites and flaviviruses, such as dengue, Zika, yellow fever, and chikungunya virus when used properly.^35^

However, there was no significant relationship between the spread of DENV to blood transfusion (*P* > 0.05). This is in disagreement with the work of Adesina and Adeniji^37^ who reported that there was statistical association between blood transfusion and transmission of DENV. Dengue virus classification as a blood pathogen has prompted a slew of studies into the virus's transfusion-related transmission. In Hong Kong, one in every 126 people is infected with DENV through blood transfusion.^38^

The tree shows that isolate D1, D5, D7, D10, D11, D12, and D14 are closely related and formed a clade with D6 and D13. Also, isolate D3 clustered with dengue virus type 2 strain 16681. Since the first case of DENV was discovered in Nigeria in 1964, DENV-2 has been the major serotype identified to be circulating in West Africa^39^ and found from sylvatic cycles in West African countries such as Cote d'Ivoire, Burkina Faso, and Guinea.^40–42^ This confirmed that the dengue virus serotype 2 isolated in our study might be imported from Thailand, where DENV-2 is known to be of wider geographical distribution.^43^

Dengue virus serotype 2 was evidenced to be the most common in West Africa, and recent outbreaks in Burkina Faso and Senegal backed up this claim. Of 700 children engaged in a hospital-based study in a semiurban area in Ghana to discover viruses that cause febrile illnesses, two were determined to be acutely infected with DENV-2 when viral RNA was detected using molecular methods.^44^ Bonney et al^45^ observed dengue virus serotype 2 from patients in Ghana suspected of having Ebola virus illness. The presence of the transmissible vector strain in the subregion may explain the dominance and circulation of Dengue virus serotype 2 in West Africa.^45^

In conclusion, it was evidence that antibodies to dengue virus are in circulation in the study population, which is also identified to be coexisting with malaria parasite. The genetic identification of dengue virus serotype 2 calls for the need to make available and strengthened preventive and control measures to curtail epidemics. The evidence of imported strains of both dengue viruses should improve/increase surveillance to imported cases.
